# A regulatory 24-bp insertion at the *BCO2* gene region is associated with yellow skin in chickens

**DOI:** 10.1186/s12711-026-01067-4

**Published:** 2026-07-12

**Authors:** Li Rong, Xueying Wu, Jiao Li, Yuting Zhou, Tong Yu, Jiuhong Nan, Liubin Yang, Xuefeng Wang, Yanping Feng, Shijun Li

**Affiliations:** 1 Hubei Hongshan Laboratory, Wuhan, 430070 China; 2 Key Laboratory of Agricultural Animal Genetics, Breeding and Reproduction, Ministry of Education, Huazhong Agricultural University, Wuhan, 430070 China; 3 The Second Affiliated Hospital & Liangzhu Laboratory, Zhejiang University School of Medicine, Hangzhou, 311121 China; 4 Key Laboratory of Smart Farming for Agricultural Animals, Ministry of Education, Huazhong Agricultural University, Wuhan, 430070 China

## Abstract

**Background:**

Chicken skin colour is an economically important trait influenced by consumer preferences across different markets. Previous studies established that white skin is dominant over yellow skin, with the β, β-carotene-9',10'-dioxygenase (*BCO2*) identified as the candidate gene. However, the precise causal mutation within this gene region has remained unidentified for over a decade, limiting the development of reliable molecular markers for breeding programs.

**Results:**

Through genome-wide association analysis of 381 chickens, we confirmed that the *BCO2* gene region on chromosome 24 was the major locus associated with skin colour. Transcriptome analysis revealed approximately 590-fold higher *BCO2* expression in white-skinned than yellow-skinned chickens. By integrating whole-genome sequencing data from 63 individuals with high-quality genome assemblies, we identified a 24-bp insertion that showed near-complete co-segregation with the yellow skin phenotype in a backcross family of 180 individuals. Electrophoretic mobility shift assays revealed an insertion-dependent DNA–protein interaction, and DNA pull-down coupled with liquid chromatography-tandem mass spectrometry (LC–MS/MS) prioritised lymphoid enhancer-binding factor 1 (LEF1) as a leading candidate interactor. Chromatin profiling revealed elevated trimethylation of histone H3 at lysine 27 (H3K27me3) and reduced chromatin accessibility at the gene locus in yellow-skinned chickens. Functional experiments revealed that knockdown of LEF1 or inhibition of enhancer of zeste homologue 2 (EZH2) significantly increased *BCO2* expression, supporting a model in which the insertion is associated with LEF1/EZH2-sensitive, polycomb repressive complex 2 (PRC2)-linked repression and transcriptional silencing.

**Conclusion:**

The 24-bp insertion in the *BCO2* regulatory region is a candidate causal variant for yellow skin in chickens, refining the genetic model of chicken skin colour variation and supporting a LEF1-PRC2-associated regulatory mechanism potentially involved in avian carotenoid metabolism. This finding enables the development of a reliable PCR-based diagnostic marker for skin colour, facilitating marker-assisted selection in poultry breeding and providing new insights into the genetic regulation of avian pigmentation.

**Supplementary Information:**

The online version contains supplementary material available at 10.1186/s12711-026-01067-4.

## Background

Chicken skin colour varies significantly between populations, which has important implications for consumer preferences and market economics [1, 2]. The genetic basis of this colour difference was revealed in early genetic studies: the white skin phenotype is completely dominant over the yellow skin phenotype, following a single-gene inheritance pattern [3, 4]. Subsequent work mapped this trait to the *W* locus, where the dominant *W* allele confers white skin through pigment degradation, whereas the recessive *w* allele permits carotenoid accumulation, resulting in yellow pigmentation [5, 6]. Building upon this classical framework, recent genomic advances have identified the molecular basis underlying this phenotypic variation. Eriksson et al. demonstrated that β, β-carotene-9′,10′-dioxygenase (*BCO2*) colocalizes with the classical *W* locus on chromosome 24 and is expressed at significantly higher levels in white-skinned chickens than in yellow-skinned chickens [7]. Mechanistically, *BCO2* catalyzes the asymmetric cleavage of carotenoid substrates (lutein, zeaxanthin), converting pigmented xanthophylls into colourless apo-10′-carotenoids [8, 9]. This enzymatic activity explains how increased *BCO2* expression leads to carotenoid depletion and the resulting white skin phenotype. Additionally, phylogenetic analyses suggest that yellow skin alleles introgressed from grey junglefowl into domestic chickens, indicating complex evolutionary origins of this trait [7, 10].

Regulatory mutations in cis-acting elements are increasingly recognized as major contributors to phenotypic diversity in domestic animals [11, 12]. These mutations often exert their effects by modulating chromatin accessibility and transcription factor binding, thereby altering gene expression patterns without affecting protein structure. A critical mechanism for establishing tissue-specific gene silencing involves the interplay between transcription factors and chromatin-modifying complexes. Among these factors, lymphoid enhancer-binding factor 1 (LEF1), a key effector of the Wnt/β-catenin signalling pathway, has emerged as an important regulator of developmental gene expression [13, 14]. Intriguingly, LEF1 exhibits context-dependent dual functionality: in the presence of β-catenin, LEF1 acts as a transcriptional activator, whereas in its absence, LEF1 can recruit transcriptional repressors, including members of the Polycomb Repressive Complex 2 (PRC2), to silence target genes [15–19]. PRC2, with Enhancer of Zeste Homologue 2 (EZH2) as its catalytic subunit, deposits the repressive histone mark H3K27me3 (histone H3 lysine 27 trimethylation), leading to chromatin compaction and transcriptional silencing [20, 21]. This LEF1-PRC2 regulatory axis has been implicated in various developmental processes in mammals, including cell fate determination, stem cell maintenance, and tissue-specific gene regulation [22, 23]. However, whether this mechanism operates in avian species to regulate pigmentation-related genes remains unexplored.

Despite significant progress in characterizing *BCO2* function and origin, the precise causal mutation remains unidentified, and practical molecular tools for routine breeding applications are still limited. Current studies have revealed that SNP (single nucleotide polymorphism) markers are strongly associated with skin colour phenotypes [24, 25]. However, these associations remain correlative and are often population specific, and the functional variants underlying the observed phenotypes have yet to be experimentally validated. Eriksson et al. (2008) narrowed the causal region to a 23.8-kb haplotype encompassing the *BCO2* regulatory region, yet the identity of the candidate causal mutation—whether single nucleotide variants (SNVs), structural variants (SVs), or other mutations affecting regulatory elements—has remained elusive for over a decade [7]. Identifying true causal mutations, whether SNVs or SVs, would not only clarify the molecular mechanisms driving carotenoid metabolism in avian species but also enable the development of reliable diagnostic markers for breeding programs and facilitate marker-assisted selection across diverse genetic backgrounds.

In this study, through the integration of multi-omics analyses (genome-wide association study [GWAS], RNA sequencing [RNA-seq], assay for transposase-accessible chromatin using sequencing [ATAC-seq], and cleavage under targets and tagmentation [CUT&Tag]) and molecular interaction experiments (electrophoretic mobility shift assay [EMSA], DNA pull-down, and liquid chromatography-tandem mass spectrometry [LC–MS/MS]), we identified a 24-bp insertion in the regulatory region of *BCO2* that is present in yellow-skinned populations as the most credible candidate causal variant within the *W* locus. We further show that the insertion is associated with an allele-specific DNA–protein interaction and with a repressive chromatin state at the *BCO2* gene region. Together with perturbation experiments targeting *LEF1* and *EZH2*, these data support a model in which the insertion facilitates LEF1/PRC2-associated repression of *BCO2*. This finding refines the genetic model of skin colour variation in chickens established over a century ago and provides a molecular foundation for precise genetic selection in poultry breeding.

## Methods

### Mapping population and GWAS analysis

To identify genetic variants underlying chicken skin pigmentation, we performed a genome-wide association study (GWAS) using a mixed linear model (MLM) approach. The mapping population consisted of 381 individuals (247 yellow-skinned and 134 white-skinned), including 213 backcross family (BCF) progeny and 168 Jinghong (JH) parent hens (Additional file 1, Table S1). All animal procedures were approved by the Animal Management and Ethics Committee of Huazhong Agricultural University (Approval Number: HZAUCH-2014-001). The BCF was established by crossing one wild-type white-skinned rooster (local Chinese breed, *WW* genotype) with 22 mutant yellow-skinned JH hens (*ww* genotype), producing 29 first-generation hens (*Ww*) that were subsequently backcrossed to the progenitor rooster to generate 213 s-generation offspring. All individuals were genotyped using a 600 K SNP array (Aviagen Ltd., Midlothian, UK) [26]. To control for population stratification between second-generation BCF progeny and JH parental chickens, as well as potential sex-related effects, sex and the first five principal components (PCs) obtained from principal component analysis (PCA) of the genotype data were included as covariates in the model, effectively minimizing false-positive associations arising from demographic confounding factors.

Different chicken populations were strategically selected for different analyses based on their suitability for each experimental objective. The BCF and JH populations were used for GWAS to ensure sufficient sample size and controlled allele segregation. For transcriptomic and epigenetic profiling (RNA-seq, ATAC-seq, CUT&Tag), Houdan (WW) and White Leghorn (ww) chickens with fixed, contrasting genotypes were chosen to maximize phenotypic contrast and reduce heterozygote complexity. WGS was performed across six diverse populations to identify variants consistently associated with skin colour across different genetic backgrounds. This multi-population design enables cross-validation of findings and reduces the risk of identifying false associations or population-specific bias.

### Logistic regression and effect size estimation

For each of the four candidate SNPs identified in the GWAS, we performed logistic regression to assess their association with skin colour (yellow vs. white, coded as 1 and 0, respectively). All the models were adjusted for sex and the first five principal components to control for population stratification. The phenotypic variance explained (PVE) by each SNP was calculated as the difference in Nagelkerke's pseudo-R^2^ between the full model (SNP + covariates) and the reduced model (covariates only), and odds ratios (ORs) with 95% confidence intervals were derived from the regression coefficients. The combined PVE was estimated by including all four SNPs simultaneously in the full model.

### Transcriptome analysis and gene expression validation

To investigate the molecular mechanisms underlying skin pigmentation differences, shank skin samples (including dermis and epidermis) were collected from three white-skinned Houdan (HD) chickens (*WW*) and three yellow-skinned White Leghorn (WLH) chickens (*ww*) for RNA-seq analysis. Differentially expressed genes (DEGs) were identified using the edgeR package [27] with stringent criteria of a false discovery rate (FDR) < 0.01 and a |log2-fold change|≥ 1. To validate the RNA-seq findings, quantitative real-time PCR (qRT-PCR) was performed on *BCO2* gene expression data from skin samples from six *WW* HD chickens and six WLH chickens, which represent the same breeds used in the transcriptome analysis. The chicken *β-actin* gene was used as the internal reference, and relative expression levels were calculated using the ${2}^{-\Delta \Delta \mathrm{C}\mathrm{t}}$ method [28]. All reactions were performed in triplicate. The sequences of primers used for qRT-PCR are listed in Additional file 1, Table S2.

### Whole-genome sequencing and screening of candidate variants

To identify candidate causal variants within the *BCO2* gene region, whole-genome sequencing (WGS) was performed on 63 individuals from six chicken populations (BCF, GJ [grey junglefowl], HD, JH, WC [Wenchang], and WLH; Additional file 1, Table S1). Variant screening employed a two-step strategy: (1) targeted scanning of the *BCO2* coding sequence and (2) an extended regional scan covering chromosome 24: 6,139,066–6,167,000 (GRCg6a), encompassing the entire *BCO2* gene and the 23.8-kb linkage region previously reported by Eriksson et al. [7]. LD (linkage disequilibrium) patterns within this region were analysed using LDBlockShow [29] with the “-SeleVar 2” parameter. Given the limitations of short-read sequencing in detecting structural variants (SVs), we additionally screened for SVs using four high-quality genome assemblies: two from yellow-skinned chickens (GRCg7w, GCF_016700215.2; GRCg7b, GCF_016699485.2) and two from white-skinned chickens (GRCg6a; Houdan) [30]. Multiple sequence alignments were performed by MUSCLE implemented in MEGA [31].

### ATAC-seq library preparation and sequencing

Chromatin accessibility profiling was performed on shank skin tissue from three white-shanked Houdan chickens and three yellow-shanked White Leghorn chickens. The stratum corneum was carefully excluded during dissection, and connective tissues were removed before snap freezing in liquid nitrogen.

ATAC-seq libraries were prepared by the YZSeq™ ATAC-seq Library Prep kit (A0215-012, Wuhan Yingzi Technology Co., Ltd., China) following the manufacturer's instructions with minor modifications. Briefly, tissue samples were ground to powder in liquid nitrogen, and approximately 0.1 g was incubated with YZ-A1 lysis buffer supplemented with protease inhibitor on ice for 30 min. Nuclei were collected by centrifugation, resuspended in DPBS (Dulbecco's phosphate-buffered saline), and filtered through a 40 μm cell strainer. For each sample, 50,000 nuclei were subjected to tagmentation with YZ-A1Tn5 transposase at 37 °C for 1 h. Following DNA purification, the libraries were PCR-amplified for 9 cycles by indexing primers, purified with 1.2 × KaPa beads, and assessed using an Agilent 2100 Bioanalyzer. Libraries that passed quality control were subjected to paired-end sequencing on an Illumina platform.

### ATAC-seq data processing

Approximately 414 million paired-end reads were generated (~ 69 million reads per sample). Adapter removal and quality trimming were performed by Trimmomatic v0.39 [32]. Clean reads were aligned to the GRCg7b reference genome (GCF_016699485.2) using BWA MEM v0.7.17 [33]. SAM files were sorted and converted to BAM format using SAMtools v1.19 [34]. Duplicate reads were removed using Sambamba v0.8.2 [35], and the reads were further filtered to retain only properly paired alignments with a mapping quality ≥ 30; mitochondrial reads were excluded. After filtering, approximately 182 million high-quality reads were retained (range: 21.1–61.4 million per sample). Normalized coverage tracks (counts per million, CPM) were generated by the deepTools v3.5.1 bamCoverage function [10]. Chromatin accessibility differences at the *BCO2* gene region between the white-skinned (n = 3) and yellow-skinned (n = 3) groups were evaluated using an independent samples t test.

### CUT&Tag library preparation and sequencing

To investigate chromatin modifications associated with skin pigmentation, Cleavage Under Targets and Tagmentation (CUT&Tag) assays targeting H3K27me3 and the IgG (immunoglobulin G) negative control were performed by the Hyperactive Universal CUT&Tag Assay Kit for Illumina (TD903, Vazyme Biotech Co., Ltd., Nanjing, China) according to the manufacturer's protocol with minor modifications.

Shank skin tissue was collected from two white-shanked Houdan chickens and two yellow-shanked White Leghorn chickens. The stratum corneum was carefully excluded, connective tissues were removed, and samples were snap-frozen in liquid nitrogen. Nuclei were extracted as described for ATAC-seq. Briefly, 0.1 g of pulverized tissue was incubated with precooled lysis buffer on ice for 30 min, and 20,000–50,000 nuclei per sample were collected and washed twice with wash buffer. Subsequent steps—including binding to concanavalin A-coated magnetic beads, primary antibody incubation (H3K27me3 or IgG control), secondary antibody incubation, pA/G-Tnp binding, tagmentation, DNA extraction, and library amplification—were performed according to the kit manual. Libraries were assessed by an Agilent 2100 Bioanalyzer and sequenced on an Illumina platform.

### CUT&Tag data processing

Approximately 934 million paired-end reads were generated from H3K27me3 and IgG control libraries (~ 117 million reads per sample). Raw data processing followed the same pipeline as ATAC-seq, with minor modifications to the Trimmomatic parameters [32]. After quality control and filtering, approximately 800.5 million high-quality reads were retained across all eight samples (H3K27me3: 90.8–147.9 million per sample; IgG: 65.5–93.6 million per sample).

### Electrophoretic mobility shift assay (EMSA)

EMSAs were performed to detect differences in protein binding between wild-type (WT) and mutant (MUT) sequences containing the 24-bp insertion. Shank skin samples (dermis and epidermis) were collected from chickens with the same genotypes and phenotypes as those used for RNA-seq, with three biological replicates per group. Nuclear proteins were extracted from ~ 60 mg of pulverized tissue using the Nuclear and Cytoplasmic Protein Extraction Kit (P0027, Beyotime, Shanghai, China) according to the manufacturer's instructions. Protein concentrations were determined by the BCA method.

Two double-stranded DNA probes (WT and MUT) were synthesized with both biotin-labelled and unlabelled (cold) versions (Shanghai Sangon Biotech, China). Probes were annealed by heating equimolar single-stranded oligonucleotides to 95 °C for 5 min followed by slow cooling to 25 °C. For each sample, four reactions were performed: (1) biotin-labelled probe alone (negative control); (2) biotin-labelled probe with nuclear protein; (3) biotin-labelled probe with nuclear protein and 200 × excess cold probe (self-competition); and (4) biotin-labelled probe with nuclear protein and 200 × excess cold probe of the alternate genotype (cross-competition). For EMSA, WT and MUT probes were each incubated with nuclear extracts from both white- and yellow-skinned chickens as indicated in Fig. 4. Reactions were incubated at room temperature for 20 min, resolved on nondenaturing polyacrylamide gels, transferred to nylon membranes, UV-crosslinked, and visualized by chemiluminescence (GS009, Beyotime, Shanghai, China).

### DNA pull‑down assay

To identify proteins that interact with the MUT probe, DNA pull-down assays were performed using a DNA Pull-down Kit (JKR23006A, GENECREATE, Wuhan, China) following the manufacturer's instructions with minor modifications. Briefly, magnetic beads were incubated with 300 pmol of biotin-labelled MUT probe (experimental) or unlabelled cold MUT probe (control) at room temperature for 2 h. After washing, the beads were incubated with 1800 µg of nuclear protein extracted from yellow-skinned chicken skin at 4 °C overnight. Following five washes, the bound proteins were eluted by boiling in elution buffer, separated by SDS-PAGE (sodium dodecyl sulfate–polyacrylamide gel electrophoresis), and visualized by Coomassie Brilliant Blue staining. Differential bands were excised and subjected to LC–MS/MS analysis (Novogene, Beijing, China).

### *LEF1* knockdown and quantitative PCR

Cell culture and transfection. DF1 chicken embryo fibroblasts were maintained in DMEM (Dulbecco's modified eagle medium) supplemented with 10% fetal bovine serum and 1% penicillin–streptomycin at 37 °C under 5% CO_2_. The cells were seeded in 12-well plates at 5–8 × 10^4^ cells per well and cultured for 18–24 h until they reached 60–70% confluence. Three siRNAs (small interfering RNAs) targeting chicken *LEF1* and a negative control siRNA (siRNA-NC) were synthesized by Sangon Biotech (Additional file 1, Table S3). Transfection was performed by Lipofectamine™ 3000 (Thermo Fisher Scientific, Waltham, MA, USA) according to the manufacturer’s protocol. Five groups were included: the untreated control (negative control, NC), siRNA-NC, and three LEF1-targeting siRNA groups (siRNA-A, -B, -C), each with three biological replicates.

The cells were harvested at 32 h post-transfection. Total RNA was extracted by TRIzol® and reverse-transcribed with HiScript IV RT SuperMix for qPCR (+ gDNA wiper) (Vazyme, R423-01). *LEF1* and *BCO2* mRNA levels were quantified by qPCR (primers in Additional file 1, Table S2), normalized to those of β-actin, and analysed via the ${2}^{-\Delta \Delta \mathrm{C}\mathrm{t}}$ method.

### GSK126 treatment and quantitative PCR

DF1 cells were seeded in 6-well plates and cultured to approximately 60% confluence. The cells were treated with either 7 μM GSK126 (EZH2 inhibitor; Beyotime, SC0060) or 0.1% DMSO (dimethyl sulfoxide, vehicle control) for 32 h, with three biological replicates per group. The cells were harvested at 32 h posttreatment. RNA extraction and qPCR were performed as described above.

## Results

### GWAS validation of BCO2 and identification of additional candidate loci

**Fig. 1:**
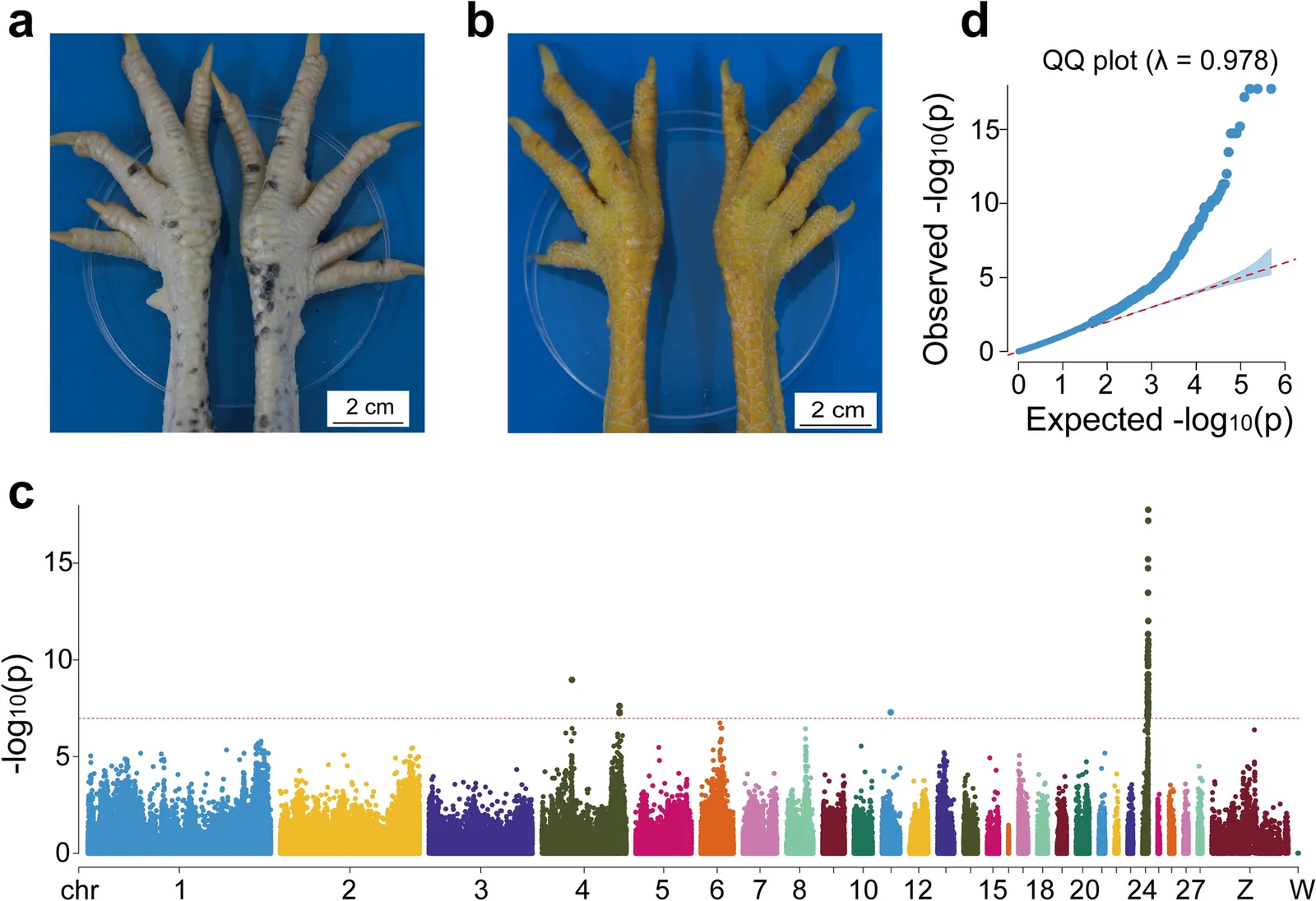
A genome-wide association study identified *BCO2* as the major locus for chicken skin colour. **a**, **b** Representative images of chicken shanks showing the two skin colour phenotypes: white-skinned Houdan chickens (**a**) and yellow-skinned White Leghorn chickens (**b**). Scale bars: 2 cm. **c** Manhattan plot of GWAS results from 381 chickens (247 yellow-skinned and 134 white-skinned) genotyped with a 600 K SNP array. The mixed linear model was adjusted for sex and the first five principal components. The red dashed line indicates the genome-wide significance threshold (*P* = 5 × 10^−8^). The lead SNP rs313221600 on chromosome 24 maps to the *BCO2* gene region. **d** Quantile–quantile (QQ) plot of observed versus expected − log_10_(*P*) values. The genomic inflation factor (λ = 0.978) indicates adequate control of population stratification

To identify yellow skin-associated variants and validate the genetic basis of skin colour variation, we performed a GWAS using a mixed linear model implemented in GEMMA. Analysis of 600 K SNP array data from 381 chickens (247 yellow-skinned vs. 134 white-skinned) confirmed that *BCO2* on chromosome 24 was the major locus associated with skin colour. The lead SNP rs313221600 (chr24: 6,143,821 bp) reached genome-wide significance (*P* = 1.78 × 10⁻^18^; Fig. 1c), had a large effect size (OR = 34.4), and explained 4.81% of the phenotypic variance. Individuals carrying at least one white-associated allele had a > 90% probability of expressing white skin, suggesting dominant inheritance, which is consistent with classical crossbreeding studies. Three additional loci reached genome-wide significance (*P* < 5 × 10^−8^; Fig. 1c; Table 1): two on chromosome 4 (rs318031896, *P* = 1.07 × 10^−9^; rs315319259, *P* = 2.40 × 10^−8^) and one on chromosome 11 (rs316901406, *P* = 5.08 × 10^−8^), each showing protective effects against white colouration (OR = 0.13–0.30). Collectively, the four significant SNPs accounted for 8.18% of the total phenotypic variance. The QQ plot (quantile–quantile plot) revealed minimal genomic inflation (λ = 0.978; Fig. 1d), indicating adequate control of population stratification.

**Table 1:** GWAS identified significant SNPs related to chicken skin colour

SNP	Chr^a^	Position (bp, galGal5)	Position (bp, galGal6)	Location	Nearest gene	P_GWAS	OR^a^	PVE^a^ (%)
rs313221600	24	6143821	6150691	Intron	*BCO2*	1.78 × 10^−18^	34.4	4.81
rs318031896	4	32193235	31939573	Intergenic	*EDNRA*	1.07 × 10^−9^	0.18	2.15
rs315319259	4	82930694	82355031	Intergenic	*ADD1*	2.40 × 10^−8^	0.3	1.77
rs316901406	11	9295288	9165791	Intergenic	*TSHZ3*	5.08 × 10^−8^	0.13	1.62

^a^*Chr* chromosome, *OR* odds ratio with 95% confidence interval, *PVE* phenotypic variance explained (Nagelkerke's pseudo-R^2^)

The mixed linear model was adjusted for sex and the first five principal components. The genome-wide significance threshold was *P* < 5 × 10^−8^. The combined PVE of all four SNPs was 8.18%

### Transcriptome analysis supports BCO2 as the primary candidate gene

**Fig. 2:**
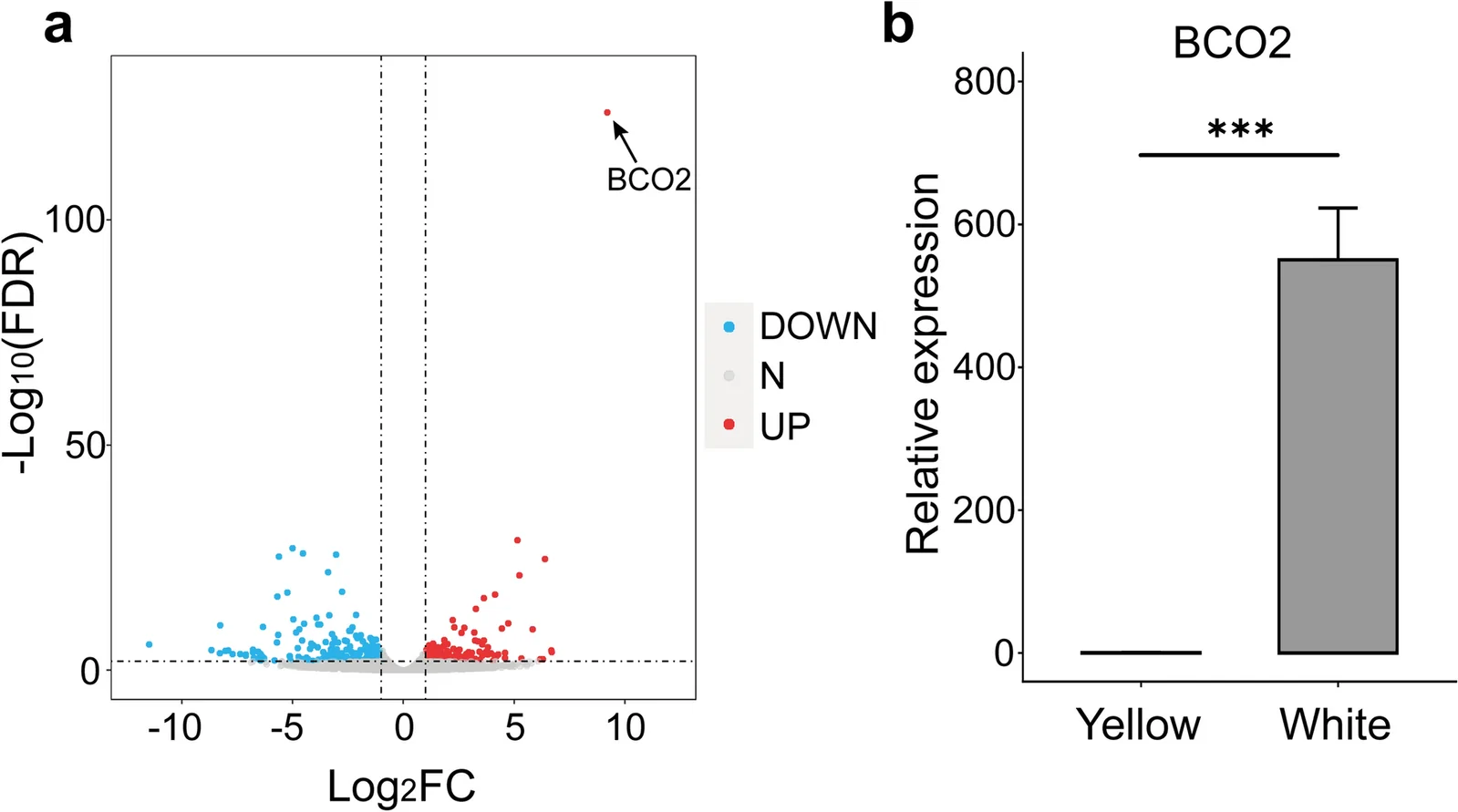
*BCO2* is significantly upregulated in white-skinned chickens. **a** Volcano plot of RNA-seq data comparing the skin transcriptomes of white- (WW) and yellow-skinned (ww) chickens (n = 3/group). The x-axis displays the log2(fold change), and the y-axis shows the significance -log10(FDR). *BCO2* (arrow) was the most significantly differentially expressed gene (log2(FC) = 9.21). The red dots represent upregulated genes, and the blue dots represent downregulated genes in white skin. **b** Validation of *BCO2* expression by qRT-PCR in an independent cohort (n = 6/group). The data are presented as the means ± SDs relative to β-actin. ***P < 0.001 (Student’s t test)

To investigate the molecular mechanisms underlying skin colour variation, we performed RNA-seq analysis on shank skin samples from three white-skinned Houdan chickens (*WW*) and three yellow-skinned White Leghorn chickens (*ww*). A total of 398 differentially expressed genes (DEGs) were identified between the two groups (Additional file 1, Table S4). Among the candidate genes located near the four significant GWAS-identified SNPs (Table 1), only *BCO2* was significantly differentially expressed. Strikingly, *BCO2* showed the most pronounced difference in the expression of all the DEGs, with markedly greater expression in white-skinned individuals (log_2_FC = 9.21, FDR = 5.50 × 10⁻^129^; Fig. 2a). This extreme fold change corresponds to an approximately 590-fold increase in *BCO2* transcript abundance in *WW* chickens compared with *ww* chickens, whereas the gene was barely detectable in yellow-skinned individuals.

We validated these findings using both quantitative and semiquantitative approaches. qRT-PCR analysis confirmed the RNA-seq results, which revealed consistent and highly significant upregulation of *BCO2* in white-skinned chickens (log_2_FC = 10.32, *P* = 3.00 × 10⁻^4^; Fig. 2b). Together, these transcriptomic results support *BCO2* as the major candidate effector gene underlying skin colour variation, with its significantly elevated expression in white-skinned chickens driving the degradation of dietary carotenoids.

### Epigenetic profiling reveals differential H3K27me3 enrichment and chromatin accessibility at the BCO2 gene region

**Fig. 3:**
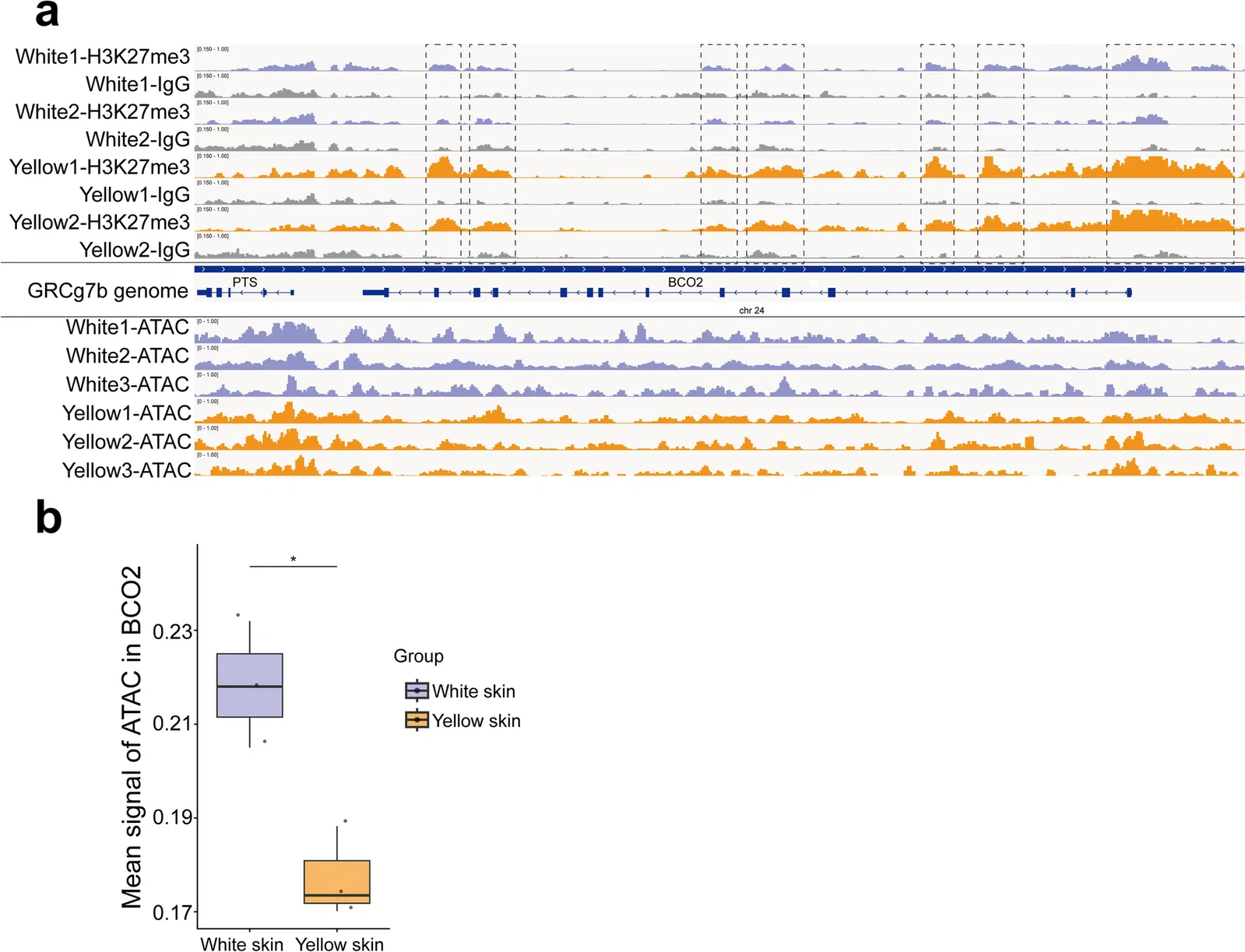
Epigenetic profiling reveals differential H3K27me3 enrichment and chromatin accessibility at the *BCO2* gene region between white- and yellow-skinned chickens. **a** Genome browser tracks at the *BCO2* gene region. Upper panel: CUT&Tag signal tracks for H3K27me3 and the IgG control in white-skinned Houdan (n = 2) and yellow-skinned White Leghorn (n = 2) chickens. The dashed boxes highlight regions with elevated H3K27me3 enrichment in yellow-skinned chickens. Lower panel: ATAC-seq signal tracks (CPM-normalized) for white-skinned Houdan (n = 3) and yellow-skinned White Leghorn (n = 3) chickens, showing differences in chromatin accessibility at the *BCO2* gene region. **b** Quantitative comparison of ATAC-seq signal intensity (CPM) across the *BCO2* gene body between the white-skinned (n = 3) and yellow-skinned (n = 3) groups. *P = 0.013, independent samples t test. The error bars represent the standard deviations

To investigate epigenetic regulation at the *BCO2* gene region, we performed CUT&Tag for H3K27me3 and ATAC-seq on skin tissues from white- and yellow-skinned chickens (Fig. 3). Genome browser visualization revealed markedly distinct H3K27me3 enrichment patterns between groups (Fig. 3a, upper panel). IgG controls confirmed the signal specificity for each sample. Notably, yellow-skinned chickens presented substantially elevated H3K27me3 signals across specific regions of *BCO2* (dashed boxes), including the promoter and gene body, suggesting that enhanced transcriptional repression was mediated by this histone modification.

Consistent with the H3K27me3 findings, ATAC-seq analysis revealed corresponding differences in chromatin accessibility (Fig. 3a, lower panel). Quantitative analysis of CPM-normalized ATAC-seq signals across the *BCO2* gene body revealed higher chromatin accessibility in white-skinned chickens than in yellow-skinned chickens (*P* = 0.013, t-test; Fig. 3b). This inverse relationship—elevated H3K27me3 coupled with reduced chromatin accessibility in yellow-skinned chickens—is consistent with H3K27me3-mediated chromatin compaction and transcriptional silencing.

Together, these epigenetic profiles provide evidence consistent with H3K27me3-associated repression of *BCO2* in yellow-skinned chickens. The coordinated increase in repressive histone marks and decrease in chromatin accessibility at the *BCO2* gene region suggest that epigenetic mechanisms are likely involved in regulating *BCO2* expression and skin pigmentation.

### Identification of a 24-bp insertion as a candidate causal variant for skin colour in chickens

To identify candidate causal variants underlying the skin colour phenotype, we utilized WGS data from 63 individuals across six populations (Additional file 1, Table S1) to screen for associated SNPs and Indels (insertions and deletions) within the *BCO2* gene. An initial scan of the coding sequence identified 9 SNPs (Additional file 1, Table S5); however, none of these SNPs could effectively discriminate between yellow and white skin phenotypes (Additional file 1, Table S6). We therefore expanded the analysis to chr24:6,139,066–6167,000 (GRCg6a), encompassing the entire *BCO2* gene and the 23.8-kb linkage region previously reported by Eriksson et al. (2008) [7]. This extended analysis identified 287 genetic variants (Additional file 1, Table S7), but despite high linkage disequilibrium (Additional file 2, Fig. S1), none completely differentiated between the two phenotypes.

Given the limitations of short-read sequencing in detecting SVs, we employed high-quality genome assemblies to systematically identify SVs within the candidate region. This approach revealed four insertion variants (10 bp, 18 bp, 24 bp, and 598 bp) exhibiting differential patterns between yellow- and white-skinned chicken breeds (Additional file 1, Table S8). Allele-specific PCR genotyping across seven populations (n = 148; Additional file 3, Fig. S2) revealed that all four insertions were fixed (frequency = 1) in the five yellow-skinned populations (Table 2). In white-skinned populations, the Houdan breed presented the complete absence of all four insertions, whereas the Wenshi-white line presented variable frequencies: the 18 bp and 24 bp insertions presented a frequency of 0.5 because all sampled individuals were heterozygous, whereas the 10 bp and 598 bp insertions presented higher frequencies (0.6), indicating incomplete associations with white skin.

**Table 2:** Allele frequency of the 4 insertion variants in the yellow- and white-skinned chicken populations

Breed	n	10 bp insertion	18 bp insertion	24 bp insertion	598 bp insertion
*Yellow skin*					
JH	24	1	1	1	1
WC	24	1	1	1	1
Yellow feather broilers	24	1	1	1	1
Hy-line Brown	24	1	1	1	1
Wenshi-yellow skin line	11	1	1	1	1
*White skin*					
Houdan	24	0	0	0	0
Wenshi-white skin line	17	0.6	0.5	0.5	0.6

Both the 18 bp and 24 bp insertions showed clear phenotype differentiation at the population level across the surveyed breeds. To distinguish between these two candidates, we genotyped 180 backcross offspring (90 yellow and 90 white) from the BCF progeny, restricted to individuals with high-quality DNA, for segregation analysis (Table 3). For the 18 bp insertion, 89 of 90 yellow-skinned individuals were homozygous for the insertion allele; however, among white-skinned individuals, 52 were homozygous, and 38 were heterozygous—failing to segregate with the phenotype. In contrast, for the 24-bp insertion, 89 of the 90 yellow-skinned individuals were Ins/Ins and one was Ins/WT, whereas among the 90 white-skinned individuals, 86 were Ins/WT and 4 were WT/WT, with no white-skinned individuals carrying the Ins/Ins genotype. Thus, among the tested variants, only the 24-bp insertion showed near-complete co-segregation with skin colour in the backcross family, with the only discordant individual being a yellow-skinned heterozygote.

**Table 3:** Genotype distribution of 18/24-bp insertions in the skin colour backcross family

Variant	Phenotype	n	Ins/Ins^a^	Ins/WT^a^	WT/WT^a^
18-bp insertion	Yellow skin	90	89	1	0
	White skin	90	52	38	0
24-bp insertion	Yellow skin	90	89	1	0
	White skin	90	0	86	4

^a^*Ins/Ins* homozygous for the insertion allele, *Ins/WT* heterozygous, *WT/WT* homozygous wild-type (no insertion)

Genomic annotation based on the chicken GRCg6a reference genome (GCF_000002315.6) revealed that this 24-bp insertion (NC006111.5:g.6154446_6154447insACATATATATATATATATATATAT) is located within the first intron of the *BCO2* gene (transcript: XM_417929.6), 6139 bp downstream of exon 1 and 1578 bp upstream of exon 2, approximately 6324 bp downstream of the transcription start site (TSS).

### The 24-bp insertion creates a novel transcription factor-binding site

To investigate whether the 24-bp insertion introduces a novel transcription factor-binding site, we performed EMSA using nuclear proteins extracted from the skin tissues of yellow- and white-skinned chickens (n = 3 per group). Two double-stranded DNA probes were designed: a wild-type (WT) probe and a mutant (MUT) probe containing the 24-bp insertion sequence.

**Fig. 4:**
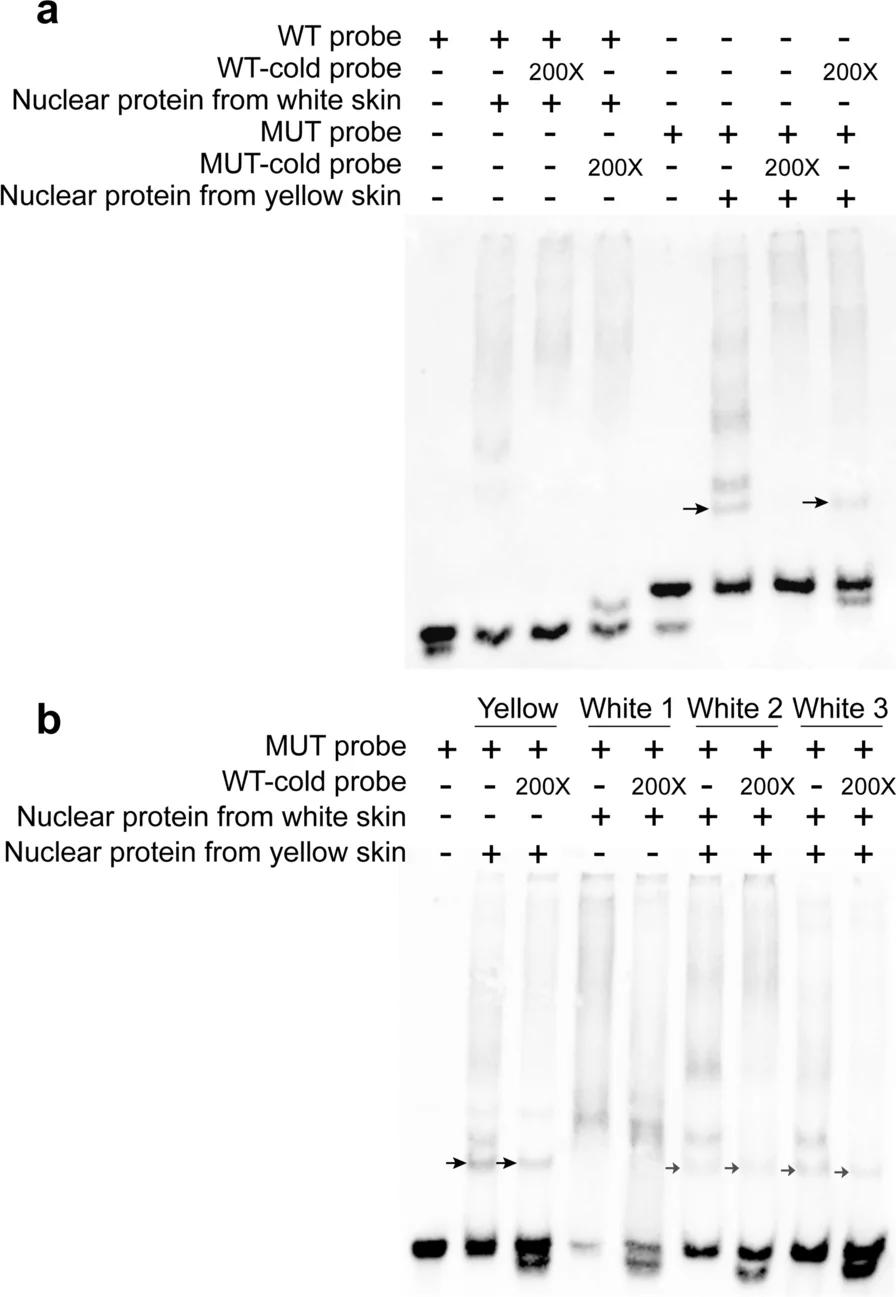
The 24-bp insertion creates a novel transcription factor-binding site, as demonstrated by EMSA. **a** EMSA of nuclear protein extracts from white-skinned (lanes 1–4) and yellow-skinned (lanes 5–8) chicken skin tissues (n = 3 per group). WT, wild-type probe; MUT, mutant probe containing the 24-bp insertion. Lanes 1 and 5, free probe (no protein); lanes 2 and 6, probe with nuclear protein; lanes 3 and 7, probe with nuclear protein plus 200 × excess self-competitor (unlabelled cold probe); lanes 4 and 8, probe with nuclear protein plus 200 × excess cross-competitor. The arrows indicate specific DNA–protein complexes observed only with the MUT probe. **b** EMSA demonstrating that nuclear proteins from both yellow- and white-skinned chickens bind the MUT probe. Lanes 2–3, yellow-skinned nuclear protein (positive control); the remaining lanes, nuclear proteins from three individual white-skinned chickens (White 1, White 2, White 3) incubated with the MUT probe. Compared with the 200 × excess WT cold probe, the shifted bands were not abolished, confirming the binding specificity of the probe to the 24-bp insertion sequence. The arrows indicate specific shifted bands

As shown in Fig. 4a, when the WT probe was incubated with nuclear proteins from white-skinned chickens, no specific DNA–protein complex was detected (lanes 2–4). In contrast, incubation of the MUT probe with nuclear proteins from yellow-skinned chickens produced a distinct shifted band (indicated by arrows, lanes 6 and 8), which was abolished by competition with 200 × excess unlabelled MUT cold probe (lane 7), confirming the specificity of this interaction. These results indicate that the 24-bp insertion sequence enables an additional sequence-dependent DNA–protein interaction that is not observed with the wild-type probe under our assay conditions.

Since no protein binding was observed with the WT probe or white-skin nuclear proteins, we next examined whether nuclear proteins from white-skinned chickens could also bind to the MUT probe. As shown in Fig. 4b, using nuclear proteins from yellow-skinned chickens as a positive control (lanes 2–3), we tested nuclear proteins from three white-skinned individuals. Notably, White 2 and White 3 showed specific binding to the MUT probe (indicated by arrows), whereas White 1 exhibited no detectable binding. Importantly, the shifted bands in White 2 and White 3 remained present upon the addition of 200 × excess WT cold probe, indicating that the wild-type sequence lacking the 24-bp insertion cannot effectively compete for transcription factor binding. This finding further confirms that the observed protein-DNA interaction is specific to the 24-bp insertion sequence. These results suggest that at least some nuclear factor(s) capable of recognising the 24-bp insertion are present in extracts from both phenotypic groups. The lack of a detectable shift in one white-skinned individual (White 1) may reflect biological variability or technical variation in nuclear extract quality. Overall, the EMSA patterns are consistent with the presence/absence of the 24-bp insertion being a key determinant of binding, rather than requiring a strictly phenotype-specific availability of the binding factor(s).

### Identification of candidate transcription factors by DNA pull-down and mass spectrometry

To identify the transcription factor(s) responsible for the specific shifted band observed by EMSA, we performed DNA pull-down assays. As shown in Additional file 4, Fig. S3, a distinct protein band of approximately 40–50 kDa was observed. This band was excised and subjected to liquid chromatography-tandem mass spectrometry (LC–MS/MS) analysis. Through molecular weight filtering, 154 candidate transcription factors were identified (Additional file 1, Table S9).

Given our transcriptomic and epigenomic data suggesting that this transcription factor functions as a transcriptional repressor of adjacent genes, we focused on candidates with known repressive activity. Among the 154 candidates, LEF1 was prioritised as a leading candidate. In some contexts, LEF1 has been reported to interact with repressive complexes including PRC2, leading to the transcriptional repression of nearby genes. Notably, the primary function of PRC2 is to catalyze H3K27me3, a repressive histone mark, which is consistent with our epigenomic findings. Notably, the pull-down/MS approach is not sufficient to prove direct binding, but it provides an independent line of evidence to prioritise LEF1 among candidate insertion-binding proteins.

### Functional validation of LEF1 and EZH2 in BCO2 regulation

To test whether LEF1 contributes to the insertion-dependent DNA–protein complex, we performed supershift assays using an anti-LEF1 antibody (ab137872, Abcam). As shown in Additional file 5, Fig. S4, no obvious supershift was observed. The lack of a clear supershift signal may reflect limited antibody cross-reactivity in chicken; therefore, direct in vivo binding of LEF1 at this locus remains to be established, although multiple independent lines of evidence prioritise LEF1 as a candidate regulator.

**Fig. 5:**
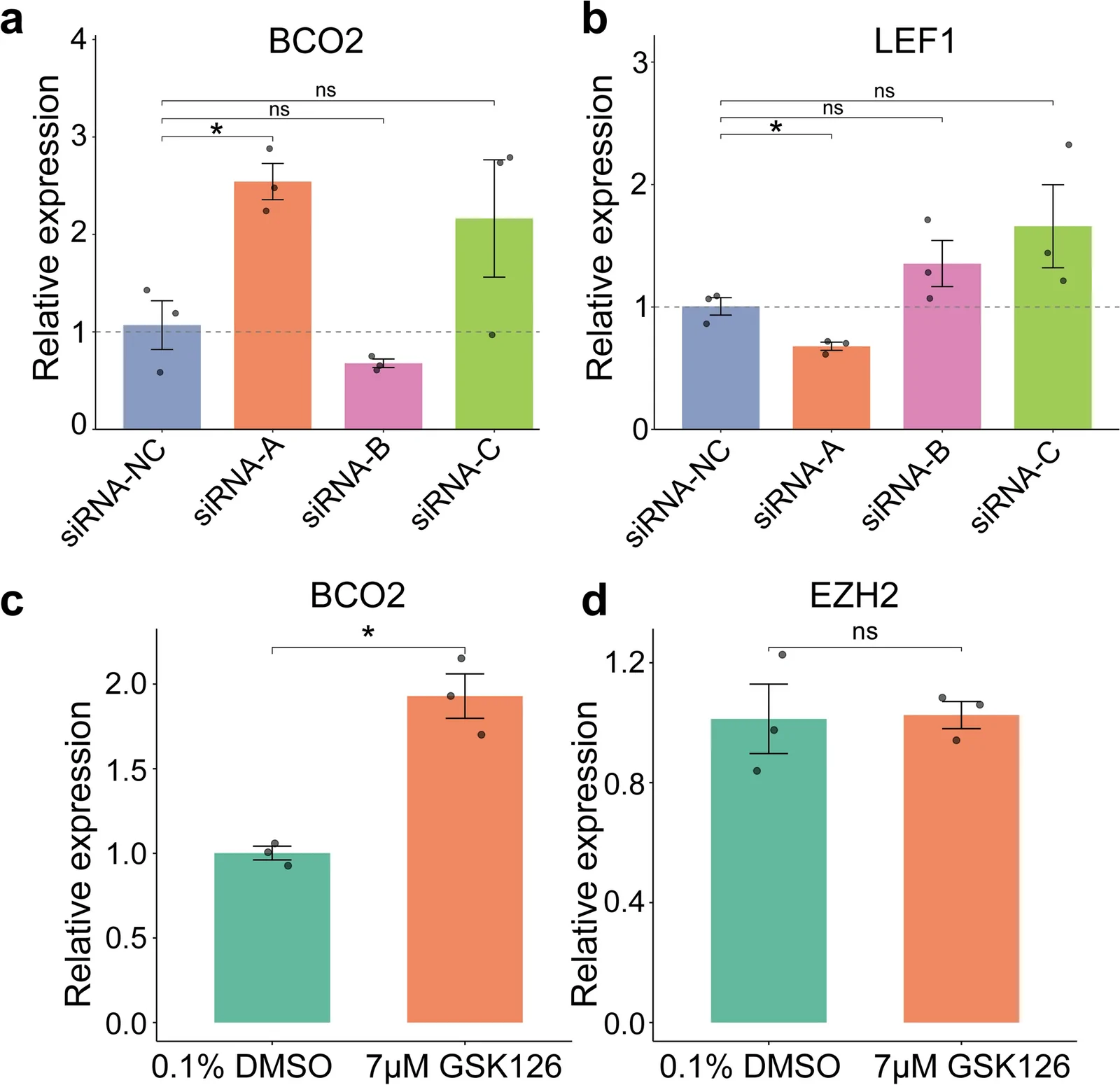
Functional validation of *LEF1* and *EZH2* in the regulation of *BCO2* expression. **a** Relative *BCO2* mRNA expression in DF1 cells following *LEF1* knockdown. Three siRNAs (siRNA-A, -B, and -C) targeting chicken *LEF1* were transfected, and gene expression was measured by qPCR at 32 h post-transfection. Compared with siRNA-NC, siRNA-A significantly increased *BCO2* expression (~ 2.5-fold). **b** *LEF1* knockdown efficiency. Compared with siRNA-NC, siRNA-A reduced *LEF1* mRNA levels by approximately 30–35%. **c** Relative *BCO2* mRNA expression in DF1 cells treated with 7 μM GSK126 (EZH2 inhibitor) or the DMSO vehicle control for 32 h. GSK126 treatment significantly increased *BCO2* expression by approximately 1.9-fold. **d** *EZH2* mRNA expression levels were not different between the GSK126-treated and DMSO control groups, which is consistent with GSK126 functioning as a competitive inhibitor of EZH2 enzymatic activity. NC, untreated control; siRNA-NC, negative control siRNA. The expression levels were normalized to those of β-actin and calculated via the ${2}^{-\Delta \Delta \mathrm{C}\mathrm{t}}$ method. *P* < 0.05 (Student's t test); ns, not significant. The error bars represent the standard deviations from three biological replicates

To further validate the role of LEF1 in regulating *BCO2* expression, we performed RNA interference (RNAi) experiments in DF1 chicken embryo fibroblasts. Three siRNAs targeting chicken *LEF1* (siRNA-A, -B, and -C) were transfected, and gene expression was assessed by qPCR at 32 h post-transfection. As shown in Fig. 5b, compared with the negative control (siRNA-NC), siRNA-A significantly reduced *LEF1* mRNA levels by approximately 30–35% (*P* < 0.05). Correspondingly, this knockdown of *LEF1* resulted in a significant increase in *BCO2* expression (~ 2.5-fold, *P* < 0.05; Fig. 5a). In contrast, neither siRNA-B nor siRNA-C produced statistically significant changes in *LEF1* or *BCO2* expression levels compared with those in the control group. Notably, only one of the three siRNAs achieved a measurable reduction in *LEF1* mRNA, which may explain the inconsistent effects on *BCO2* expression across siRNAs.

To investigate the involvement of the PRC2 complex in this regulatory mechanism, we treated DF1 cells with GSK126, a selective inhibitor of EZH2, the catalytic subunit of PRC2 responsible for H3K27me3 deposition. As shown in Fig. 5c, treatment with 7 μM GSK126 for 32 h significantly increased *BCO2* expression by approximately 1.9-fold compared with the DMSO vehicle control (*P* < 0.05). Notably, the *EZH2* mRNA levels did not differ between the GSK126-treated and control groups (Fig. 5d, *P* > 0.05), which is expected since GSK126 functions as a competitive inhibitor of EZH2 enzymatic activity rather than as a suppressor of its transcription.

Taken together, these findings support a model in which the 24-bp insertion is linked to LEF1-associated repression and PRC2/EZH2-dependent H3K27me3 deposition at the *BCO2* gene region, thereby reducing chromatin accessibility and *BCO2* transcription.

## Discussion

Skin colour is a classic Mendelian trait in chickens that has been studied for more than a century. Here, we provide convergent evidence that a 24-bp insertion in a *BCO2* intronic regulatory region is a strong candidate causal variant associated with yellow skin in chickens based on multi-omics analyses, SV screening, and molecular interaction experiments. We performed a GWAS using 600 K SNP array data from 381 chickens and confirmed that *BCO2* on chromosome 24 was the major locus associated with skin colour. The lead SNP (rs313221600) had a remarkably large effect size (OR = 34.4), explaining 4.81% of the phenotypic variance. Through whole-genome sequencing of 63 individuals and high-quality genome assembly comparisons, we identified a 24-bp insertion that showed near-complete co-segregation with the yellow skin phenotype in a backcross family of 180 individuals. We propose that the 24-bp insertion acts as a cis-regulatory element that may facilitate allele-specific engagement of a repressive regulatory machinery. EMSA indicates an insertion-dependent DNA–protein interaction, and pull-down/MS prioritises LEF1 as a candidate interactor. Consistently, yellow-skinned chickens show increased H3K27me3 and reduced chromatin accessibility at the *BCO2* gene region, and *BCO2* transcription can be partially de-repressed by *LEF1* knockdown or pharmacological inhibition of EZH2. Together, these convergent lines of evidence support a LEF1/PRC2-linked model for *BCO2* silencing associated with the 24-bp insertion. Notably, although different cohorts were used for GWAS, transcriptomics, epigenomics, and segregation analysis, all independently converged on the 24-bp insertion and *BCO2* repression, indicating that our findings are robust and not an artefact of any single population.

Importantly, the segregation pattern of the 24-bp insertion in the backcross family was near-complete rather than absolute. Under the proposed dominance model, white skin is expected in both Ins/WT and WT/WT individuals, whereas yellow skin is expected primarily in Ins/Ins individuals. Accordingly, the four white-skinned WT/WT birds are consistent with expectation, and the only truly discordant case was a single yellow-skinned Ins/WT individual. Although this rare exception does not change the overall pattern of association, it means that the current evidence, while strongly supportive, does not by itself constitute definitive proof of causality. This discordance may reflect phenotypic misclassification, incomplete penetrance, or the effects of modifier loci, consistent with the suggestive signals detected on chromosomes 4 and 11.

The evolutionary origin of the yellow skin allele has been a subject of considerable interest. Eriksson et al. demonstrated that yellow skin alleles introgressed from grey junglefowl into domestic chickens, representing one of the most convincing examples of adaptive introgression in domestic animals [7, 36]. Our finding that the 24-bp insertion is fixed in all five yellow-skinned populations examined, including both commercial and indigenous breeds, is consistent with this introgression hypothesis. The complete fixation of this variant across diverse yellow-skinned populations is consistent with the possibility of positive selection and/or strong breeder preference, likely driven by human preference for yellow skin colouration in certain markets. Notably, white-skinned populations presented either complete absence (Houdan) or intermediate frequency (Wenshi-white line) of this insertion, further supporting its candidate causal role in determining skin pigmentation. However, it should be noted that our study was limited primarily to commercial breeds and indigenous populations from East Asia and Europe. Further validation in chickens from other geographic regions would strengthen the generalizability of our conclusions and provide additional insights into the global distribution of this candidate causal variant.

LEF1 (lymphoid enhancer-binding factor 1) is a transcription factor belonging to the LEF1/TCF (T-cell factor) family that plays crucial roles in the Wnt/β-catenin signalling pathway. Intriguingly, LEF1 can function as either a transcriptional activator or repressor depending on its interacting partners. When β-catenin is absent, LEF1 recruits transcriptional repressors, including members of the PRC2, to silence target genes [15–19]. PRC2 is a histone methyltransferase complex with EZH2 as its catalytic subunit and is responsible for establishing the repressive H3K27me3 modification [20, 21]. Our CUT&Tag data revealed elevated H3K27me3 signals at the *BCO2* gene region in yellow-skinned chickens, which is consistent with a PRC2-linked repressive mechanism and with LEF1 as a candidate contributor.

While direct confirmation of LEF1 binding by a supershift assay was challenging, potentially owing to the limited cross-reactivity of the mammalian-derived antibody with the chicken LEF1 protein, the subsequent perturbation experiments are consistent with LEF1/EZH2-sensitive regulation of *BCO2*, although they do not by themselves establish direct binding at this locus. Specifically, siRNA-mediated knockdown of *LEF1* significantly increased *BCO2* expression, whereas GSK126 treatment (an EZH2 inhibitor) also upregulated *BCO2* without affecting *EZH2* expression levels, suggesting that EZH2 enzymatic activity contributes to *BCO2* repression in this cellular context. The magnitude of *LEF1* knockdown varied across siRNAs; future work using CRISPR-based perturbation or additional validated reagents will be helpful to further strengthen the causal link. Although DF1 fibroblasts do not fully recapitulate the skin cellular context, the consistent de-repression of *BCO2* upon *LEF1* or *EZH2* perturbation provides functional support for a repressive regulatory mechanism. These findings support a model in which the 24-bp insertion introduces an allele-specific protein-binding site, with LEF1 prioritised as a candidate factor that may facilitate PRC2-dependent H3K27me3 enrichment and transcriptional repression of *BCO2*. In the context of pigmentation, LEF1/TCF factors have been shown to regulate melanocyte differentiation and pigment gene expression in mice and humans through interactions with chromatin modifiers [37–39]. Our findings suggest that the LEF1-PRC2 axis represents a conserved regulatory module that has been coopted for carotenoid-based pigmentation in avian species, warranting further cross-species comparative studies to explore the evolutionary conservation of this mechanism.

The interactions between transcription factors and chromatin modifiers play a critical role in establishing tissue-specific gene expression patterns. Our ATAC-seq analysis revealed significantly greater chromatin accessibility at the *BCO2* gene region in white-skinned chickens than in yellow-skinned chickens (*P* = 0.013), which correlates with the dramatically higher *BCO2* expression observed in white-skinned individuals (approximately 590-fold). This finding is consistent with our proposed model: in yellow-skinned chickens, the 24-bp insertion may enable binding of one or more nuclear factors (with LEF1 prioritised as a candidate), which in turn may facilitate PRC2-linked H3K27me3 enrichment, reduced chromatin accessibility, and repression of *BCO2*. Conversely, in white-skinned chickens lacking this insertion, the repressive machinery may be less efficiently recruited, resulting in an open chromatin state and high *BCO2* expression. Notably, our EMSA experiments demonstrated that the transcription factor(s) capable of recognizing the 24-bp insertion sequence are present in nuclear extracts from both yellow- and white-skinned chickens. These EMSA results suggest that the nuclear factor(s) capable of recognising the 24-bp insertion are present in both phenotypic groups, arguing that the key difference is the presence/absence of the insertion sequence rather than an absolute requirement for phenotype-specific availability of the binding factor. This finding is particularly important for understanding the molecular mechanism: the insertion creates the binding site rather than altering the expression of the cognate transcription factor.

*BCO2* encodes β, β-carotene-9′,10′-dioxygenase, an enzyme that catalyzes the asymmetric cleavage of carotenoid substrates, including lutein and zeaxanthin. In white-skinned chickens, high *BCO2* expression leads to efficient degradation of dietary carotenoids, preventing their accumulation in the skin. Conversely, in yellow-skinned chickens, the near-complete silencing of *BCO2* allows carotenoids to accumulate, resulting in yellow pigmentation. This enzymatic mechanism has been well characterized in previous studies [40], and our transcriptomic data showing approximately 590-fold higher *BCO2* expression in white-skinned chickens provide strong support for this model.

Despite *BCO2* being the major locus for skin colour determination, our GWAS identified three additional loci with genome-wide significance on chromosomes 4 and 11. These SNPs had protective effects against white colour (OR = 0.13–0.30), with each explaining 1.62–2.15% of the phenotypic variance. However, none of the candidate genes near these loci were significantly differentially expressed according to our transcriptome analysis. Further investigation is needed to determine whether these represent true modifier loci or population-specific associations. The identification of potential modifier loci suggests that skin colour determination may involve a more complex genetic architecture than previously appreciated, although *BCO2* remains the predominant contributor.

The candidacy of the 24-bp insertion as a causal variant is supported by (i) near-complete co-segregation in a backcross pedigree, (ii) population-level fixation in multiple yellow-skinned lines and absence/low frequency in white-skinned lines, (iii) a near-complete loss of *BCO2* transcription in yellow skin, (iv) concordant repressive chromatin features (increased H3K27me3 and reduced accessibility), and (v) partial de-repression of *BCO2* upon perturbation of LEF1 or EZH2 activity. Establishing genotype–phenotype relationships for regulatory mutations remains challenging, particularly when the causal change lies in non-coding sequence and acts through chromatin-dependent mechanisms [41–43]. Our results narrow the long-standing W-locus signal to a specific short insertion and link this variant to allele-specific protein binding, a repressive chromatin environment at the *BCO2* gene region, and functional sensitivity to LEF1/EZH2 perturbation, collectively supporting an insertion-associated LEF1/PRC2-linked repression model for *BCO2*. Several cis-regulatory mutations have been shown to underlie phenotypic variation in chickens, including copy number variation in intron 1 of *SOX5* (Pea-comb) [44], an 8.3-kb deletion upstream of *SOX10* (Dark brown plumage) [45], a large insertion downstream of *BMP12* (Naked neck) [46], a complex rearrangement involving *EDN3* (Fibromelanosis) [47], and an EAV-HP insertion in the 5′ flanking region of *SLCO1B3* (Blue eggshell) [48]. In line with these examples, our study refines the molecular basis of yellow skin from a mapped haplotype to a candidate causal SV and provides a practical PCR-based marker for breeding applications.

The practical implications of our findings can be extended to poultry breeding programs. The identification of the 24-bp insertion provides a simple PCR-based marker for skin-colour selection. In our backcross family, all Ins/Ins individuals displayed yellow skin, and no white-skinned individual carried the Ins/Ins genotype, indicating strong practical value for marker-assisted selection (MAS) against yellow skin. Nevertheless, broader validation across additional populations and breeding backgrounds will be important to confirm the robustness of this marker.

Despite the convergent genetic, epigenomic and functional evidence presented here, several limitations should be noted. First, although the 24-bp insertion shows near-complete co-segregation with skin colour, the presence of one discordant yellow-skinned Ins/WT individual means that causality cannot yet be considered definitive from segregation evidence alone. Definitive proof will require allele-editing experiments, such as CRISPR/Cas9-mediated insertion/deletion followed by phenotyping and locus-specific chromatin assays. Second, the proposed involvement of LEF1 remains provisional: DNA pull-down/MS prioritised LEF1 and perturbations of LEF1 or EZH2 de-repressed *BCO2*, but the supershift assay was inconclusive and direct LEF1/PRC2 occupancy at the endogenous locus in skin has not yet been demonstrated. Third, functional validation was performed in DF1 fibroblasts, which may not fully recapitulate the relevant skin cellular context, and *LEF1* knockdown efficiency was modest for two of the three siRNAs. Finally, our population sampling, while diverse, does not cover the full global spectrum of chicken genetic diversity, and additional validation across broader breeds and environments is needed to confirm marker universality and to clarify the contribution of potential modifier loci detected on chromosomes 4 and 11.

## Conclusion

Overall, we identified a 24-bp insertion in the *BCO2* regulatory region as a candidate causal variant for yellow skin colouration in chickens. Multi-omics profiling and functional perturbations support a model in which this insertion is associated with a repressive chromatin state at the *BCO2* gene region, potentially mediated by LEF1/PRC2-linked mechanisms. This insertion is associated with reduced *BCO2* transcription and carotenoid accumulation. Our findings refine the genetic model of skin colour variation in chickens established over a century ago and provide a molecular foundation for precise genetic selection in poultry breeding. Further studies on the detailed mechanism of the LEF1-PRC2 interaction at this locus and the potential roles of modifier loci on chromosomes 4 and 11 will provide additional insights into the complex regulation of pigmentation in avian species.

## Supplementary Information

**Additional file 1: Table S1**. Sample information of chicken populations used in GWAS and WGS. Detailed information on the chicken populations used in this study, including breed name, sample size and data type. **Table S2**. Primer sequences for qRT-PCR and structural variation genotyping at the *BCO2* gene region. Primer sequences for quantitative real-time PCR of the *BCO2*, *LEF1*, *EZH2*, and *β-actin* genes and for genotyping four structural variants (10 bp, 18 bp, 24 bp, and 598 bp insertions) in the *BCO2* gene region. **Table S3**. siRNA sequences targeting chicken *LEF1* and negative control siRNA. Sequences of three *LEF1*-targeting siRNAs and one negative control siRNA used for RNA interference experiments in DF1 cells. **Table S4**. List of genes differentially expressed in skin tissues between yellow- and white-skinned chickens. Complete list of 398 DEGs identified by RNA-seq analysis (FDR < 0.01, |log2FC|≥ 1). **Table S5**. SNPs identified in the coding sequence of the *BCO2* gene. Nine SNPs identified within the *BCO2* coding region from WGS data of 63 individuals. **Table S6**. Association analysis between *BCO2* coding sequence SNPs and chicken skin colour phenotypes. Genotype frequencies of the nine coding SNPs across yellow- and white-skinned populations. **Table S7**. Whole list of genetic variants identified in the extended *BCO2* genomic region (chr24: 6139066–6167000, GRCg6a). All genetic variants identified in the chr24:6139066–6167000 region (GRCg6a) encompassing the *BCO2* gene and 23.8-kb linkage region. **Table S8**. Detailed information on four candidate insertion structural variants (SVs) in the *BCO2* gene region. Four insertion variants (10 bp, 18 bp, 24 bp, and 598 bp) identified by high-quality genome assembly comparison between yellow- and white-skinned chickens. **Table S9**. List of candidate transcription factors identified by LC–MS/MS with molecular weight filtering. List of 154 candidate transcription factors identified from the DNA pull-down assay, filtered by molecular weight (40–50 kDa).

**Additional file 2: Figure S1**. Linkage disequilibrium (LD) pattern within the *BCO2* candidate region. LD heatmap of the extended *BCO2* region (chr24: 6,139,066–6,167,000, GRCg6a) generated using LDBlockShow with the “-SeleVar 2” parameter. Data derived from whole-genome sequencing of 63 individuals.

**Additional file 3: Figure S2**. Allele-specific PCR genotyping of candidate structural variants. **a**–**d** Representative gel electrophoresis images of allele-specific PCR genotyping for the four candidate insertion variants (10 bp, 18 bp, 24 bp, and 598 bp). The PCR products were resolved on agarose gels to distinguish the insertion and wild-type alleles. In each gel image, lanes 1–3 represent wild-type chickens, whereas lanes 4–6 represent mutant chickens.

**Additional file 4: Figure S3**. A DNA pull-down assay identifies candidate transcription factors that bind to the 24-bp insertion sequence. **a**, **b** SDS-PAGE analysis of proteins captured by DNA pull-down using a biotin-labelled MUT probe (with a 24-bp insertion) or an unlabelled cold probe (control). Nuclear proteins were extracted from yellow-skinned chicken skin. The same gel is shown as the original colour image (**a**) and the grayscale image for enhanced visualization (**b**). The red arrows indicate a distinct band (~ 40–50 kDa) specific to the MUT probe, which was excised for LC-MS/MS analysis.

**Additional file 5: Figure S4**. Supershift assay with an anti-LEF1 antibody. MSA supershift assay using anti-LEF1 antibody (ab137872; Abcam). Lane 1: free MUT probe; Lane 2: MUT probe with nuclear protein from yellow-skinned chickens; Lane 3: MUT probe with nuclear protein plus anti-LEF1 antibody. No obvious supershift was observed, potentially due to the limited cross-reactivity of the mammalian-derived antibody with the chicken LEF1 protein.

## Data Availability

The datasets supporting the conclusions of this study are available in the Genome Sequence Archive (GSA, https://ngdc.cncb.ac.cn/gsa) repository, CRA020638 (transcriptome data), CRA020642 (epigenomic data) and CRA020724 (genome data); SNP array data are available in the OMIX repository, No. OMIX007999 (https://ngdc.cncb.ac.cn/omix) [49].
